# Weaving rapport: doctors’ strategies towards patients’ noncompliance

**DOI:** 10.1186/s12913-022-08947-7

**Published:** 2023-01-05

**Authors:** Yinong Tian, Jing Zhang, Haiyang Che, Yonggang Su

**Affiliations:** 1 School of Foreign Languages and Literature, Shandong University, Jinan, Shandong China; 2 Orthopedics Department, Zibo Maternal and Child Health Care Hospital, Zibo, Shandong China

**Keywords:** Doctor-patient discourse, Rapport management model, Noncompliance

## Abstract

**Background:**

A successful therapeutic rapport between doctors and patients is built on effective doctor-patient communication. Noncompliance of patients which challenges their communication has been described in the research, yet the rapport strategies are not well discussed.

**Methods:**

This qualitative study investigates the rapport strategies when doctors face noncompliance in consultations and its pragmatic effects achieved through the doctors’ speeches. The 10-hour recordings come from the doctor-patient communication in the hospital setting. Thereafter, we analyze their conversation following the Spencer Oatey’s rapport management model.

**Results:**

Compliments and joking in the illocutionary domain, storytelling in the discourse domain, the doctors’ participation in the participation domain and the choice of appropriate titles in the stylistic domain are identified and analyzed as the rapport-building strategies.

**Conclusion:**

The present study has offered insights into physicians’ rapport-building strategies in the face of rapport-threatening behavior from patients. These strategies will help the doctors to deal with rapport-challenging behavior and boost overall patient wellness.

## Background

The interaction that occurs between doctors and patients is the art of medicine. Doctors perform as suppliers of services, while patients are consumers [1]. Doctors gather healthcare needs and physical information from patients to make an accurate diagnosis and deliver treatment advice to patients. Healthcare providers, including doctors in hospitals, shape or reinforce a patient’s outcomes through statements in medical consultations. Relief can often be achieved with the adoption of certain statements [2] associated with mutual understanding. Careful consideration of words by doctors can reduce levels of anxiety among patients [3], and when doctors acknowledge their emotional cues and respond empathically, they actually alleviate a feeling of vulnerability in patients [4]. Besides, this type of response has a positive influence on patient satisfaction and adherence to treatment plans [5]. It is useful to convert patients’ objections into acceptances of treatment recommendations through doctors’ statements [6]. They will act as a dynamic duo if consultations go as planned. However, influenced by limited consultation time and other factors, that very often the reverse is the case. Conflicts between doctors and patients are inevitable, resulting in a possible rapport collapse. In hospital departments the tendency of doctors is to ignore and avoid these conflicts [7], which actually impedes their effective communication and affects rapport. While prior studies have confirmed that employing good strategies improves patients’ compliance [8] and enhances their relationship [9], the rapport between doctors and patients is still an untapped research area in pragmatics and conversation analysis. With limited communication skills, doctors commonly feel overwhelmed to construct a good relationship with patients in out-patient clinics. To explore this premise, this study is conducted to analyze the rapport management strategies in doctor-patient consultations. In particular, it intends to uncover how physicians manage and build rapport when patients show rapport-threatening behaviors. Besides, it also answers the pragmatic effects achieved in the process of alleviating noncompliance. In what follows we first review prior literature from two aspects, including doctor-patient discourse and rapport management. Drawing from the above two research questions, we next discuss the doctors’ rapport-building strategies from four domains in Rapport Management Model (RMM). Throughout we rely on a rich background of literature as well as on data collected across several months. The contributions in this study are twofold: (1) an analysis of rapport-threatening behavior from patients in the four domains; (2) a series of doctors’ strategies associated with rapport-threatening behavior built from that analysis.

## Doctor-patient discourse

In health care, the doctor-patient discourse is an essential dynamics, affecting the course of patient compliance with treatment. Unlike other types of discourses, it has been studied using differing approaches.. Pragmatics seeks to account for the rules that govern the use of language in these contexts [10]. Many pragmatic researches aim to uncover the medical processes alongside their solutions. Among the dominant themes within medical discourse is asymmetry. A number of researches describe the asymmetry condition from physicians’ genders [11], topic and task asymmetry [12], the medical agenda [13], initiative asymmetry [14], medical interview asymmetry [15], and the remarkable persistence of asymmetry [16]. In the paternalistic relationship, this asymmetry allows doctors to control the process and keeps patients passive. Patients are given information designed to encourage them to agree with the doctor’s decisions. This description of imbalanced interaction between doctors and patients has been challenged during the last 20 years [17]. So, how to adapt to the asymmetry becomes a research focus at the same time. Taking the doctors as double agents, contractual or legal incentives [18] can be a solution to the information asymmetry. The doctor-patient communication has a more complicated nature than other interactions [19]. Therefore to further reach the equilibria needs more solutions. Back in 1999, small talk was pointed out to lessen the negative impact of this dominance [20]. In the handbook of discourse analysis, Nancy [21] mentioned that questions are usually seen as embodying asymmetry and therefore should be treated appropriately. Alex [22] stressed the importance of adaptive responses to non-ideal speech situations. Besides, the shift from information sharing to interpretation schemes sharing [23] also offered new insight into symmetry solutions. Later, AI was employed to resolve the information asymmetry [24], but it was difficult to promote this technology-driven innovation. Yan [25] concluded that mitigations could reduce the negative influence of such misalignment and relieve patients’ anxiety. A view of facilitating a relationship through shared understanding and shared decision-making was also supported in many studies, including Zhang [26], McCabe [27], Kee [28], Langberg [29], Rostoft [30], Caitríona and Zoë [31], van Dael [32]. However, these studies generally provide limited strategies for physicians to manage tough consultations. More broadly, doctors not only have to learn and use more strategies but also face even tougher challenges in the management of interpersonal relations. Therefore, it is necessary to explore this previously hidden area with frameworks related to interpersonal relations. A related framework that runs both implicitly and explicitly through interpersonal relations is the rapport management model. Rapport, a feeling of connection and closeness with one another, is clearly linked to the relationship between doctors and patients. The management of rapport reveals the strategies that have powerful effects on interpersonal relations in medical consultations. Accordingly, the Spencer-Oatey’s rapport management model will be discussed in the following section.

## Rapport management

Since the publication of Brown and Levinson’s ‘politeness’ theory in 1978, the adaptation of this theory to conversations in various settings has become a trending topic among inter-disciplinary work. Although serving as the pivotal theory, it still has attracted criticism. One of the problems is that ‘politeness’ strategies mainly cover limited types of interaction [33]. A broader perspective shows that politeness serves as a way to reduce face-threatening acts and enhance social relations, including professional conversations between patients and physicians [34]. Anchored in politeness theory, Rapport Management Model (hereafter RMM) was proposed by Helen Spencer-Oatey to study beyond the face-saving or threatening acts and move to the focus of interpersonal relations in certain settings. RMM introduces a new standpoint that there are a variety of orientations from which people approach rapport, depending on several factors. These orientations consist of rapport enhancement (to enhance a harmonious relationship), rapport maintenance (to maintain a harmonious relationship), rapport neglect (indifference to the relationship), and rapport challenge (to challenge or impair a relationship). A key feature of RMM is that the rapport management is characterized by a number of interrelated domains, including the illocutionary domain, the discourse domain, the participation domain, the stylistic domain and the non-verbal domain. The illocutionary domain is associated with speech act utterances, varying from the selection of speech components, and the extent of directness-indirectness to the choice of upgraders or downgraders. For example, M Černý [35] examined the function and the character of speech acts in medical interaction based on this domain. The role of directness/indirectness in doctor language was delineated by Pop A [36] to promote health communication. In terms of the discourse domain, relevant research aims to unearth the openings and closings in tele-homecare encounters [37] or rapport management in WhatsApp chats [38]. In the participation domain, there are investigations into the turn-taking systems [39] and inclusion/exclusion of people present, like patient participation [40]. Tone [41] and genre [42] are often discussed within the stylistic domain, and the non-verbal domain is often studied through a multi-modal approach. However, RMM strategies within these domains are only touched in few research about physician-patient consultations. Clearly, compared with other settings, there is more work to be done on RMM and rapport strategies in medical consultations. Recently years, the notion of RMM has been considered one of the best frameworks for the impoliteness analysis covering politeness research [43] and the related study of pragmatics [44]. In medical consultations, a successful therapeutic doctor-patient relationship is expected to have shared perceptions [45] regarding the nature of the problem, treatment goals, and following support. Expectations are shaped by norms in the above interrelated domains [46], which contribute to the basis of rapport strategies. Consultations encompass the professional and intricate interactions that serve as the cornerstone of their mutually beneficial relationship. Their verbal or non-verbal communication is fraught with complications concerned with different domains. So, this multifaceted approach could decode the complexities of medical consultations, and provide rapport strategies which is tailored to the medical setting. Therefore, I consider RMM particularly suitable to analyze physician-patient consultations as this framework, focusing on the rapport management strategies used by physicians in the interaction dynamics. When patients’ behaviors affect this dynamics and damage the rapport, it is physicians’ strategies that become the game-changer in rapport management.

## Methodology

### Research questions

1. How physicians manage the rapport with patients when the latter exhibits noncompliance in consultations?
2. In the process of alleviating the above disagreements, what pragmatic effects are achieved through the doctors’ speeches?

### Data collection

Data collection is operated strictly in accordance with the Consolidated Criteria for Reporting Qualitative Studies (COREQ). In the Chinese healthcare system, doctors from out-patient departments, comparable to family doctors, play a more vital role in providing a full range of medical services. Physicians in this study are selected through convenience sampling. At first, the researcher interviewed 10 physicians working in different departments. Seven physicians refused to participate or dropped out because of scheduled appointment and operations. Therefore, three male physicians, with an average age of 33, are chosen as the participants from departments, including physical examination center, orthopaedics department and physiotherapy department. They all have already practiced their professions for more than 5 years and have extensive experience in all aspects of clinical operations. The patient participants include 55 male patients and 45 female patients from the age of 20 to 90. It includes patients who consented to be tracked in subsequent consultations after being informed of the situation in advance. To be specific, 51 patients visit the physical examination center, 29 patients expect medical instructions from the orthopaedics department and 17 patients turn to the physiotherapy department for further advice. Table 1 in the following contains the demographic profile of patients. No one else presents besides the participants and researchers in this study. Natural conversations between doctors and patients are collected from a Grade-A Tertiary Hospital in a prefecture-level city of China where doctors consult with patients to shape relevant diagnoses, initiate subsequent therapy, and establish a caring relationship. The duration of new patient consultations in outpatient settings lasts for 10 hours and these radio data are recorded between December 2021 and February 2022. The 10-hour recordings form a small corpus (of about 87,000 words) with 100 consultations varying from 3 min to 10 min each. Considering privacy requirements from patients, the researcher does not record the video data.

**Table 1:** The demographic profile of patients

		Number	Percent
Sex	Female	45	45%
	Male	55	55%
Age	≤20	1	1%
	21–40	15	15%
	41–50	12	12%
	51–60	18	18%
	61–70	30	30%
	71–80	21	21%
	81–90	3	3%
Familiarity with doctors	All new visits		

### Data analysis

The 100 consultations are transcribed according to the transcription system first developed by Gail Jefferson [47] for work in conversation analysis. Each utterance in the corpus is transcribed into Mandarin Chinese. Utterances shown in the examples are also translated into English for research. It is noted that the variety of Chinese dialects in the data may not match Standard Chinese because a large number of patients come from different districts and counties. Besides, transcription is not a once-for-all-time representation of talk [48] but rather an open-ended process with refinement. Therefore, the researcher frequently re-transcribes the data to distinguish nuances of accents and improve transcription accuracy. To ensure that the transcript is consistent with the original recording, another researcher is also trained to check 90% of the transcript which is randomly selected; reliability is 95%. Transcripts are returned to participants for comment and correction before the final version. The analysis is accompanied and supported by RMM to uncover doctors’ rapport strategies towards rapport-threatening behavior. Since video data is not included in the corpus, four interrelated domains, excluded the non-verbal domain, are discussed in this study. According to RMM, the four interrelated domains consisted of the following domains, illocutionary domain (performance of speech acts), discourse domain(discourse content and structure), participation domain(procedural aspects such as turn-taking and inclusion/exclusion of people present), stylistic domain(e.g., tone, genre) [49]. Referring to a large proportion of work on the physician-patient relationship, rapport management was rarely analyzed. Even for those studies mentioned the domains [50–52], they stopped short of further discussions into the conditions that how speakers took use of rapport management strategies to maintain or improve communication. In this study, themes within four interrelated domains and a set of rapport-building strategies used by doctors are identified and derived from the radio data. These strategies are especially pointed for the condition when doctors have to deal with patients’ rapport-threatening behavior in consultations. It is noted that there is consistency between the radio data presented and the findings, and the four major domains as well as a description of diverse cases are clearly presented in the following findings.

## Research findings

This section highlights doctors’ strategies for enhancing rapport from four interrelated domains of RMM and uncovers pragmatic effects achieved through doctors’ speeches. The performance of speech acts, including a range of various components, can be rapport-enhancing or rapport-threatening [53]. A patient who refuses to follow doctors’ advice with excuses posed a challenge or threat to the doctor-patient consultation process and treatment results. In the face of such rapport-threatening behavior, a doctor often adopts rapport management strategies along with rapport-enhancing behavior to promote health care delivery. In this connection, the study first extends this line of research by analyzing the doctor-patient rapport in four domains and explores their rapport management strategies for effective communication and better health outcomes.

### Illocutionary domain

Compared with other domains, most research space is devoted to the illocutionary domain [49]. The illocutionary domain, first drawn heavily by Brown and Levinson [53], concerns the rapport-threatening or rapport-enhancing implications of performing speech acts. The illocutionary domain covers widely, such as apologies, requests, compliments, gratitude, and so on [46]. The appropriate handling of speech acts is crucial for the establishment of harmonious communication relations [54]. In typical healthcare settings involving a doctor, a patient, and their utterance, there are many kinds of acts in various domains of RMM. Doctors in our study show a preference for constructing rapport through compliments and joking. According to previous studies, compliments typically positively effect on interpersonal relations by offering support or approval [53]. For instance, as indicated in Extract 1, the doctor complimented the patient’s physical fitness in a general health check.

Extract 1.

**Table:** 

1.	D:	平时这个走路啊腿酸麻胀痛吗? Do you feel painful or aching and tingling or numb when you walk?
2.		(1.5)
3.	P:	不疼 No, not really.
4.	D:	不疼吭现在还能出去遛弯吗? Um. Can you still go out for a walk now?
5.		就这八十多了= Since you are over eighty=
6.	P:	=还能溜这溜啊还能太极呢 =I often stroll around the neighborhood and play tai-chi.
7.		啥事没有不用拐杖 I’m fine without crutches.
8.	D:	=那那那很好啊(hhenhh) =It’s cool(hhenhh)
9.		您这是宝刀未老啊 You are really a treasure knife which does not age.
10.		行行啊没事 Okay, okay. It’s all right.
11.	P:	好啊 Yippee.
12.	D:	昂 Well.
13.		(0.3)
14.		你现在现在整体状况整体状况很好啊 Your overall situation is really good.
15.	C:	°是吧 ° °I think so°
16.	D:	他现在呢就是主要就是这个就是到了这个年龄啊↑ Now, he has aged into eighty.
17.		就是出去遛弯: So, it is especially important to take care of him while out for walks:
18.		(1.0)
19.		注意防止摔倒. Look out for falls and fall prevention.
20.		以后出去啊一定拿着呃拿着拐杖= Never leave home without a waking aid, a crutch=
21.	P:	=拐杖(hhenhh) = =The crutch (hhenhh)=
22.	D:	=对对对 =Exactly! No doubt about it!
23.		一定拿着拐杖 Make sure to take a crutch
24.		(1.0)
25.	P:	那拐着怕丢人 I am afraid of losing face and embarrassing myself
26.		(.)
27.	D:	呃#你这八十多了呵呵:你这八十多岁了你: Um#You are over eighty:come on, you are over eighty years old!
27.		要是腿磕一下 If got a nasty crack on the leg
29.		还敢不拿拐杖吗(hhenhh) Dare you not take a crutch(hhenhh)
30.		虽然还有另外一条腿呢 Although you still have another strong leg
31.		但是咱也不能学丹顶鹤啊(hhenhh) You can’t live like a red-crowned crane(hhenhh)
32.	P:	哈哈哈(hhenhh) Ha ha ha(hhenhh)
33.		放心吧一定照做= Don’t worry. I will do that.
34.	D:	=哈哈哈(hhenhh) Ha ha ha(hhenhh)

Compliments in medical consultation are frequently used to enhance mutual understanding among participants and strengthen emotional exchanges for the preparation of further communication [55]. In Extract 1, the patient has a regular health check, and the medical report shows that he is in a better physical state than older adults of the same age. Here, the doctor responds by first affirming the patient’s fitness at this age, then by complimenting the patient’s statement that he still practices tai-chi (an internal Chinese martial art) despite being over eighty and no longer needs the crutch, and finally by assessing his capability to walk (‘It’s cool(hhenhh)’;line 7). In the opinion of the patient, he wants to be appreciated for such a physical condition, which marks the need for respect. In terms of the patient’s extrovert personality and desires, the doctor immediately responds with an idiom (‘You are really a treasured knife which does not age’; line 8) as a way of compliment. In ancient Chinese literature called the Romance of Three Kingdoms, this idiom refers to old people who are still capable and enthusiastic about participating in activities as they did in the past. It is prevalent in Chinese society and has seen ages of constant use [56]. This idiom not only plays a social function of improving the patient’s confidence but also shows consideration for the patient’s respectability. Note, in line 10, the patient immediately accepts the physicians’ words (‘Yippee’), indicating a positive mood and his preference to receive the doctor’s further affirmation rather than making excuses. As a continuation of the conversation, the doctor repeats compliments using different expressions (‘Your overall situation is really good; line 13). At this time, the patient also provides a positive assessment of his own health as illustrated in line 14. In Extract 1, It becomes evident that the patient takes an equal part in the relationship and maintains rapport in the follow-up consultation. As Wolfson stated, one of the functions of compliments is to encourage the desired behavior [57]. Older patients are expected to be occupied with their improved mental and physical state. The respectability required of this patient extends to the degree of congruence between judgments of his condition, including his physical fitness. Since compliments can be identified as face-threatening, the doctor chooses appropriate words instead of the highly exaggerated compliment content to establish common ground with this stubborn patient and reach the rapport goal.

Another related subcategory of rapport-enhancing behavior is joking. Joking, as a part of the humor, finds a natural home in conversation. The joking activity, a double-edged sword, plays an important role in rapport management. Joking is expressed in specific terms that refer to specific people [58], such as doctors, nurses, and patients, and their identities in the medical setting. Hospitals are shrouded in constant pain and complaining, increasing the likelihood of exhaustion [59] among healthcare workers and patients. Therefore, medical consultations are in great need of joking strategies to enliven patients and create a joyful atmosphere. Extract 1 exemplifies a sequence of talk with a joke. Upon seeing the patient’s embarrassment over using a crutch and his excuses (line 25), the doctor realizes that he should help the patient feel at ease and assure the patient that crutches are not stupid (line 27). In accordance with earlier studies, on similar occasions, many physicians prefer to perform rapport-sensitive speech acts, including requests or orders [60], to encourage better communication. However, the doctors’ orders are usually loaded with power and position [61], which might threaten equity rights and even arouse irritation or annoyance. In this particular case, the physician in Extract 1 presents the importance of using crutches in a humorous and relaxing way by joking, which has rarely been discussed in the past. The breadth of usage of joking is demonstrated in many ways, including greeting or leaving-taking, introducing various topics or participants, relieving tension, covering embarrassment, and filling in awkward moments [62]. Since the patient feels embarrassed and has no actual recourse to handle these feelings, the aim of a joker, that is, the physician here is to produce positive reactions. This is to dispel embarrassment, dissolve excuses, and establish a positive rapport. For the physician in Extract 1, powerless in the face of his obdurate patient, humor, like joking, might be one of the rapport-building ways to cope. Generally speaking, patients are less often the target of jokes than symptoms; if jokes are about patients, it is more often those who have “brought on their own medical problems” [63]. The joke targets at the high risk of cracking on the patient’s legs and the patient’s resistance to crunches. The physician first points out that the serious consequence is the loss of one leg, with laughter in line 28. The laughing could be interpreted as a way to release the built-up tension. After that, the physician makes exaggerations that the patient might possibly become “a red-crowned crane” (line 30) which often stands on only one leg if he falls down and still refuses to use crunches. Through this act, the doctor hands the decision-making power to the patient, which is equity placement. The patient shrieks with laughter, confirming that he follows the physician’s advice to use crunches in line 32. To respond to the patient’s feedback, the physician laughs with cohesion (see the equals sign in line 32 and line 33). Since they finally get a release from this talk and are able to manage the relationship without frustration and annoyance, they have been laughing together for a while. Both the doctor and the patient have different degrees of “cost and benefit” in this extract. Equilibrium has been calibrated through joking and laughter in a light-hearted way. As a result, achieving equal rights and a good rapport comes naturally. Overall, the doctor uses compliments or jokes to help the patient feel confident that he would not lose face if he walked with a crutch. The impromptu use of an idiom leads to a change in the patient’s attitude towards crunches, and laughter also helps to decrease the patient’s anxiety. Compliments and jokies in the illocutionary domain have the underappreciated benefit of resolving communication problems, carrying implications for interpersonal relations, and offering helpful insights into rapport management strategies.

### Discourse domain

The production and understanding of the discourse domain derive from discourse structures [64], such as topic choice and management, and the sequence organization of information. Among studies, the sequence organization is of primary importance because social interactions are characterized by a sequential occurrence of social actions [46]. In medical settings, the sequence organization flows from the concern with the doctors’ strategies toward the patient’s feedback. Medical settings organize sequences by starting with the doctor’s strategies and ending with the patient’s feedback. Analyses of sequences in this study indicate that storytelling is commonly used in consultations, serving as a rapport-building “lubricant”. In new patient visits, when patients display their understanding of symptoms, the floor is yielded, allowing physicians to check diagnostic x-rays and other factors that may contribute to the symptoms. However, patients are least cooperative with treatment recommendations [65] when they desire more treatments than they receive from physicians and often make excuses to stick to their earlier lifestyles. In Extract 2, a patient adopts active resistance to negotiate a new treatment that aligns with her thoughts.

**Table:** 

1.	D:	血压高啊你看都160了 Your blood pressure is up to 160 now.
2.		(1.5)
3.	P:	不高啊就是偶尔才160 It’s as high as 160 only sometimes.
4.	D:	hh.你走路的时候脚疼不疼啊= hh. Feet hurt when walking?
5.	P:	=而且我早上起来、午睡后、晚上睡前测得都正常啊 Numbers are normal when I checked in the morning, at noon and before bedtime.
6.		就是看到白大褂紧张了 It is you in the white coat that leads to the higher number.
7.	D:	哈哈哈哈 (laughing)
8.		(4.0)
9.	P:	我脚::我脚上贴着膏药. Feet::Plaster cast on my feet.
10.	D:	哦贴着膏药哈,因为我一看你的膝盖好像有点变形啊 Whoa, plaster cast. It seems that the knee shape has changed.
11.	P:	我感觉还行啊 I feel good.
12.	D:	你这个大姨就是说-如果啥的话想治的话: Aunt, I mean-if you want to heal the knee injury:
13.		你得查一下, It’s better to check the cause in detail.
14.		我妈也是这样= My mom had the same condition=
15.		=后来拖着就不行了哎 =It dragged on and finally did not pull through.
16.		孩子是疼在心里也是没辙了 My heart aches but there is really no way.
17.	P:	哎 So sorry.
18.	D:	我作为孩子辈实在见太多这种例子了 I have seen too many cases as a child and doctor.
19.		吭::就特别你这个膝盖,我因为我-你一进来-我看到膝盖变形了. ow::your knees are particularly..because I see its crooked shape as soon as you walked in.
20.		还是尽快做个核磁共振看看吧 It’s better to have MRI as soon as possible.
21.		你看前面几个身体不如你都好了 Several are not as good as you, and all fully restore to health.
22.		身子骨能好得更快 You will recover sooner.
23.		医保也能报销啊 The health insurance also covers it.
24.	P:	行吧 Okay.
25.	D:	提前治.不然到后期啊你要再变形再-然后再厉害了 Early intervention works. Otherwise, it will become more serious later.
26.		你难受孩子也心疼啊 Your child also feel distressed when you are sick.
27.		到时候就得-换-换膝盖了 knee replacement is the choice when it is far worse=
28.	P:	=吓人啊那肯定很疼啊 That’s so scary and painful.
29.		行我就听你的! Fine, I follow your treatment plan.

As illustrated in line 5, the patient begins her explanation with a gentle presentation of her normal blood pressure and then proceeds to ascribe her irregular blood pressure to the doctor’s white coat (line 6), which is called white coat hypertension [66]. Clearly, this explanation aims to free herself from whole responsibility for uncontrolled high blood pressure. The doctor senses that the time of spotting her explanation may not be precisely right, and that their rapport may be at risk. So, instead of directly giving an authoritative answer, the physician laughs in line 7 to fix this awkward situation. Thereafter, their talk comes to a halt, lasting for 4.0 s. It is, therefore, the doctor’s responsibility to ensure that the patient receives timely and appropriate treatment within a harmonious rapport. In response to the doctor’s previous question regarding foot pain (line 4), the patient first mentions her plaster cast on the feet (line 9). This sudden change of topic interrupts the physician’s thinking. In addition to her words, the doctor looks at her from feet to knees, and the crooked shape of her knees. The doctor points out the crooked knees, which is likely an underlying symptom of high blood pressure. Despite his direct dissuasion, the doctor uses little authority, and this speech act actually prevents potential negative consequences and establishes a harmonious rapport. It is supported by the patient’s indirect response, which shows no resistance beyond repeating her physical condition. So far in this sequence, the doctor gains an accurate understanding of her “literally hard mouth”, and decides to use storytelling as a strategy. In order to make his emotional involvement interpretable to her, the doctor skillfully tells a fact-based story through the inclusion of a personal topic (line 14) about what happened to his mom. The story’s climax is not delivered in a neutral tone or manner. Rather, it is reinforced by the doctor’s sharing of his mother’s physical suffering (line 15) and his experience of powerlessness and helplessness, although he is a physician (line 16). In practice, the story about the doctor’s mother is a clear cue that may or may not be a continuum of the patient. There is a marked difference between this persuasion and other persuasions in this story. There is a marked difference between this persuasion and other persuasions in this story. Besides, empathy is often created along with storytelling, serving as the engine of rapport management [67]. It is clear from the patient’s voice that the doctor’s empathy is fairly consistent, as she signs with sadness in a tense, pressed voice. Later, the doctor describes her knees’ crookedness (line 19) and recommends magnetic resonance imaging (MRI) as a treatment in line 20. The storyteller proceeds to introduce cases of recovered patients and the doctor assures she can even recover sooner under treatments. In subsequent consultations about the high cost of magnetic resonance imaging, the doctor informs that MRI is already covered in free public healthcare. The patient’s antipathy is relieved when she replies, avoiding any potential rapport-threatening consequences. Even so, the patient still does not directly agree with the doctor’s treatment. In this case, shortly afterward the doctor encourages the patient to weigh up different options between early intervention and more painful symptoms (line 25) following previous history takings. Concomitantly, the patient displays astonishment and affiliative agreement of the painful experience in a high pitch register(see line 28 and exclamatory mark in line 29). The doctor thus receives the patient’s permission for further medical treatment. In Extract 2, the articulation of other patients’ stories, and the process of putting words to the experience by physicians allow patients to position themselves as insiders and even allies to these stories. It is not only the verbal storytelling that increases emotional involvement, but also the way that the doctor repeats the pain feeling even in more or less the same way. It is through the sharing of stories that physicians are able to examine and critique the lives of others, which allows patients to understand their own positions and have reflexivity throughout the treatment process. According to patient feedback, storytelling has been found to be effective for gaining emotional information [68] and heightening emotional engagement when a patient challenges rapport. Through the interpretation of empathy and emotional involvement in the story, rapport is established and built, even if it is challenged at the beginning of consultations. The storyteller, therefore, upgrades the displays and rendering of emotionswhen the doctor’s advice does not match the patient’s feelings and thoughts, allowing the patient to actively participate in the rapport.

### Participation domain

Compared with other domains, the participant domain receives limited attention regarding RMM. It deals with the procedural aspects of interchanges [46] that have unlimited contexts. A key aspect of the participation domain is the inclusion and exclusion of people present. Participation in medical consultations refers to the joint efforts of doctors and patients who exchange information, share medical expertise, build an enhanced rapport, and ultimately make health-related decisions together. In fact, medical treatment satisfaction and control over patients’ health are closely associated with doctors’ participation. As evidenced in the following data, doctors’ participation plays a significant role in rapport management and treatment acceptance, particularly when patients engage in rapport-challenging behaviors. For instance, in Extract 3, an elderly patient with high blood pressure visits a doctor for the first time in the department of cardiology.

**Table:** 

1.	D:	如果不吃药啊,很危险. No drugs will definitely put you into danger.
2.	P:	这这这十盒药都要三百啊 But..but..but a ten-box supply of medicines costs three hundred Yuan
3.		长期吃哪行啊 No, it is not allowed to eat it for a long time.
4.	D:	是是不过大姨咱们吃国产的一样啊 Yes, I know it is a burden. But domestic drugs have the same curative effect.
5.		只要能降压就行 As long as it can lower your blood pressure.
6.		吭,脑溢血就麻烦了. This time, however, it could all go wrong with cerebral haemorrhage.
7.	P:	我还是挺正常的,就这两年 I am fine in recent two years.
8.	D:	吭大姨作为大夫来说,你整体很好. Ahh. I could give you a a clean bill of health.
9.		咱现在条件好,咱不是说没钱啊,每个月拿着这么多退休金 We have a good living condition now. You also enjoy the sufficient pension.
10.		咱得好好活着啊.多看看小孙子小孙女啊 Live your life. Spend more time with your grandchildren.
11.		不能说我不把这个东西当回事儿, You cannot disregard for life.
12.		大姨你说,我高的时候一百五, As you say, its only one hundred high.
13.		我低的时候一百三十多,这个就不用管了. It is only one hundred and thirteen low and therefore we can dispense with it.
14.		你就不用吃药了. Then you don’t have to take medicine.
15.		但是你现在是什么问题啊, But the point is that,
16.		高的时候一百六七了,这就不行了. the blood pressure is as high as one hundred sixty or seventy. It’s risky.
17.	P:	也是啊,俺还要看小孙女嘞 Well, I’m blessed to watch my granddaughter grow up.
18.		身体不能不行. Fitness is foremost.

In Extract 4, a senior patient with cerebrovascular diseases has a consultation with a physician after a thorough physical examination.

**Table:** 

1.	D:	对对对,因为您这个年龄啊,咱一定要睡眠质量好 Oh, yeah, yeah. Must ensure you get enough good-quality sleep.
2.		不然的话,咱这个心脑血管儿这个疾病的风险= Otherwise, the risk of cardiovascular and cerebrovascular diseases is=
3.	P:	=我去年吃安定了感觉没事了啊 =I ate some antipsychotic last year and that’s fine.
4.	D:	啊对对对,你吃安定这不是个长[法儿 Yes, yes. Antipsychotic is not a long-term solution.
5.		安定吃多了还会骨折啊 Antipsychotic could cause fractures.
6.		这个年纪可摔不起啊 You can not afford to fall down at this age.
7.	P:	也是啊一把年纪了 Well, I am getting on a bit now.

As shown above, patients initially refuse to follow their physicians’ treatment advice at first, attempting to challenge their harmonious rapport (see line2 and line 7 in Extract 3, line 3 in Extract 4). Patients’ words imply that they believe they should not be unduly controlled or imposed upon treatment recommendations. This kind of behavioral expectation can be different in some ways, like how it affects the way patients think in this setting. However, it is obviously not really ethical, and this act is against doctors’ duties and responsibilities. Direct medical advice is inherently face-threatening, and doctors ought to select appropriate rapport strategies to mitigate the face-threat and be polite. In Extract 3, the doctor first deals with issues of concern by prescribing alternative medicines in line 4 and mentions the same pharmaceutical effect even if the patient uses the medicine at a lower price. Then, the doctor tells the patient that she is a “skip-generation” grandparent to show how important it is for her to spend time with her grandchildren. The majority of Chinese older people are much emotionally attached to their grandchildren than to their own children. Thus, the doctor’s argument is sure to be emotionally touching since the patient is likely to accept the advice in order to spend more time with the children. In Extract 4, the doctor mentions the serious consequences of taking antipsychotics over long periods of time and highlights that the patient is of a certain age now. Considering his age, the patient finally accepts the doctor’s professional advice and moves on to his later life. In both extracts, after the doctors’ participation, patients demonstrate more active attitudes as well as a heightened commitment to healthcare activities. The patients’ involvement in consultations, coupled with the physicians’ participation, creates a circle of rapport. Due to patients’ behaviors that pose a threat to the doctor-patient relationship, it is evident from the proceeding conversation that doctors devote significant effort to rapport management. The doctors’ participation, as a dynamic process, goes beyond providing professional medical advice, displaying empathy and strengthening the therapeutic alliance. Rather, it consists of a range of patient-centered rapport strategies, such as creating a variety of opportunities for mutual gain, offering a number of options and expressing emotions. In other words, rrgardless of how conscientious a physician may be, his capability will be impractical if he cannot associate himself with a patient and establish close rapport. In general, a mutually trusting and respectful rapport is a hallmark of joint participation, especially the doctors’ participation, in which patients accept timely medical treatments and doctors have higher job satisfaction as well as enhanced self-esteem. When patients disregard the presence of doctors’ treatment advice to shatter the rapport, it is the doctors’ participation that cures both diseases and their rapport.

### Stylistic domain

Typically, three key components are typically discussed within the stylistic domain, including choice of tone, use of genre-appropriate lexis and syntax, and use of genre-appropriate honorifics and titles. Despite the fact that researchers generally agree on the importance of the stylistic domain, it is still typically discussed from the patients’ perspective rather than doctors. In pragmatics, the role of genre-appropriate honorifics or titles in medical consultations is gradually becoming clear [69]. Despite the fact that researchers generally agree on the significance of the stylistic domain, it is typically discussed from the perspective of patients rather than physicians. In pragmatics, the role of genre-appropriate honorifics or titles in medical consultations is gradually becoming clear [69]. In this study, the major rapport-building strategy identified within this domain is the choice of appropriate titles that demonstrate esteem, courtesy or respect. During patient-centered consultations, doctors usually greet patients at the openings and establish concordance as well as rapport connection. The use of an appropriate title in greeting creates a positive first impression, and this simple action helps both parties find their niches and develop into their roles. The titles with a high frequency and/or wide range of occurrence across the data are the “laoshi”(老师in Chinese, laoshi in pinyin), “auntie” (大姨in Chinese, dayi in pinyin) and “uncle”(大叔in Chinese, daye in pinyin). The polite title “auntie” in this context, refers to the oldest maternal aunt or the sister-in-law. In Extract 6, the doctor initiates a motivated sequence by calling an elderly female patient an auntie, as the confirmation of her status. Thus, the doctor absorbs the patient into his own circles and asks her about the medical and surgical history smoothly. It then comes as a pleasant surprise that the patient in the follow-up shows continued cooperation including compliance with prescribed regimens, which reflects the rapport function of polite titles. The line down to the right represents the hidden content which is not vital for the analysis here.

**Table:** 

1.	D:	来,大姨坐到凳子上. Come on, auntie, seat down please.
2.	P:	哦,好::: Ah, fine:::
3.		(4.0)
4.	P:	谢谢啊, Oh, thank you.
5.	D:	行,大姨. Okay, auntie.

The title “uncle” performs a similar function but has distinct meanings, referring to the father’s elder brother or uncle. In Extract 7, the patient sometimes feels numbness, pain and tingling in the legs, and therefore comes to this hospital for treatment recommendations. The doctor greets the patient by calling him “uncle”, allowing the patient to perceive himself as a full participant with attachment and closeness from the start. “The first blow is half the battle.” Naturally, the general interrogation starts in an unruffled way and an accomplished rapport naturally with ease.

**Table:** 

1	D:	来,大叔坐到凳子上. Come on, uncle, seat here please.
2.		(清嗓子) (clear throat)
3.		(4.0)
4.		平时有手麻或者头晕吗? Have you usually experienced hand numbness or dizzy?
5.	P:	没有. Nope.
6.	P:	好好,谢谢. Alright, thanks.
7.	D:	哎,没事儿. That’s no problem.

The doctor in Extract 7 uses a respectful tone to call the male patient “uncle”, thereby elevating the patient’s status and narrows the psychological distance. The consultation’s middle section consists of a comprehensive history and physical examination, which minimizes his risk of developing a serious illness. The use of “uncle”, stretching through this consultation, is exhibited by the physician without thinking about it. As part of the final treatment, the patient exhibits less anxiety regarding his symptoms and greater tolerance for over-the-counter medicine of prescription strength. With these title-based speech acts, this patient enters the consultation with a positive attitude, establishing a good rapport and eliciting better treatment outcomes. In general, the extension of kinship, including the above titles, socializes the non-kin patients into family relationships. The use of these titles by the doctor introduces more politeness into the interpersonal dynamics and promotes greater harmony in rapport. The third title is the “laoshier” in Chinese pinyin. As a dialect, it serves as a title that exceeds the meaning of “teacher” in English. It was once regarded as a title of respect for teachers, and gradually grows into a term referring to individuals who impart knowledge or have qualities others should learn from [70]. As a gender-neutral title, it is commonly used in central Shandong to address individuals of any profession, especially when asking strangers to open the door for further conversation [71]. The use of “liaoshier” flows across the whole corpus. Take Extract 8 as an example.

**Table:** 

1.	D:	对.然后老师您这个呢,先::不要乱做按摩. That’s right. Laoshi, you would better now::don’t take a massage.
2.		吭钱也没少花. It really, cost a bundle.
3.	D:	吭,咱不能说老师您这儿::整天这治那治,现在 Yet, we can not::try medication here and there, now
4.		目前来说,就是效果不好. For now, treatment effect is not good.
5.	D:	行老师,建议做核磁,不着急. Okay, laoshi. I suggest nuclear magnetic resonance, not in a rush.
6.		您这个回去看看. Take a moment to think about it.
7.		咱说看清楚了,咱咱说怎么治. Get familiar with your case, and then turn to the treatment plan.
8.	P:	嗯.Hum.
9.	D:	对对对.Yes.

In Extract 8, the patient initiates discussion of inappropriate and irregular self-medication. This case prohibits the doctor from expressing his opinion. Therefore, he tries to break the ice by addressing the patient as “liaoshier” (line 6 and 8). The doctor then sits in the driver’s seat to initiate a new sequence. In addition to mentioning the preferred treatment in line 10, the doctor also refers to the patient as “liaoshier” again. To prevent the patient from feeling judged, the doctor here always responds politely with a respectful title “liaoshier” whenever the patient provides a challenging response. Therefore, the patient feels encouraged to express satisfaction with the recommended treatment. At the end of their consultation, the patient complies with the physician’s instructions and accepts the full treatment plan, indicating a harmonious rapport building. Starting conversations with titles, including “laoshi”, “auntie” and “uncle” is equivalent to the recognition of patients’ status and the presentation of doctors’ politeness and respect. The doctors treat their patients as if they were their own family members and insist on delivering medical advice with courtesy during consultations. To make appropriate use of titles, doctors have to master the dimensions of use. It is also essential to identify the pragmatic conditions for using titles to denote varying degrees of politeness. For example, the specific use of titles varies based on the status, age, and gender of patients. Since patients seem quite aware of correlations of power or status in medical consultations, doctors should learn how they are expected to call male or female patients in consultations. To conclude, titles, especially with a sign of respect, are also noteworthy for a doctor-patient rapport in medical contexts.

## Discussion

**Fig. 1:**
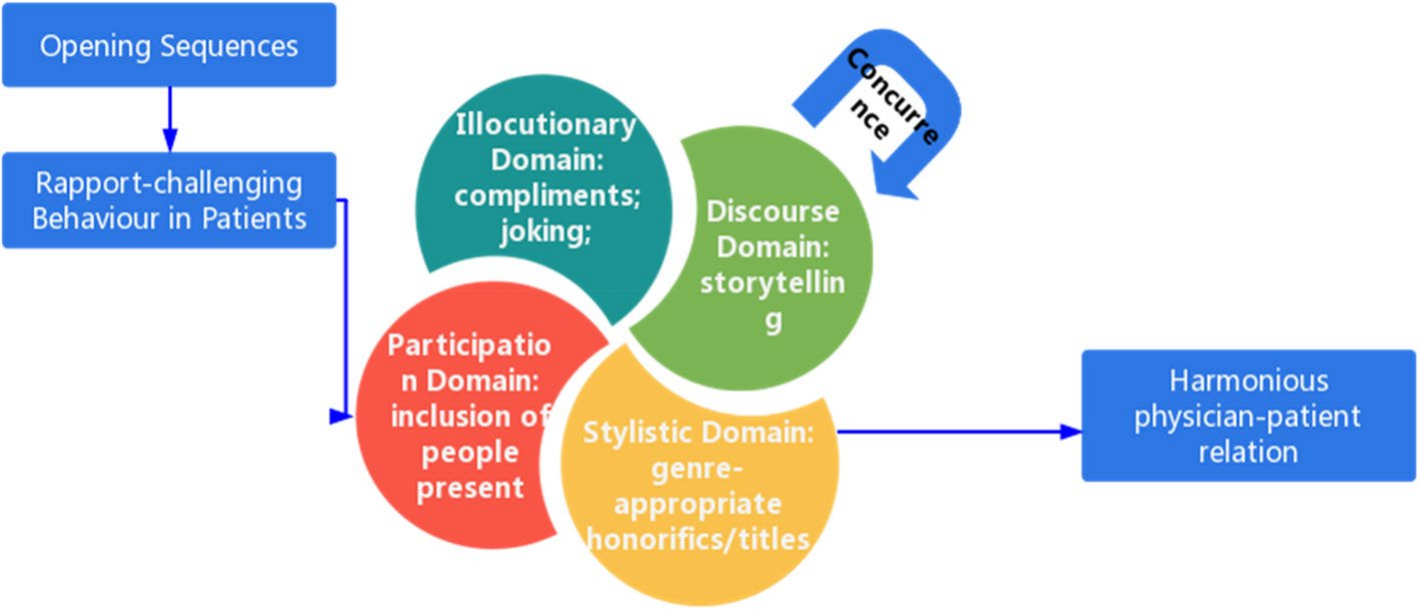
A rapport management model between doctors and patients in outpatient clinics

Effective consultation between doctors and patients is at the heart of patient-centered care. Of the various strategies that contribute to an effective consultation, certainly one of the most important is the ability to perform rapport management in front of rapport-challenging behavior. As we shall see, the failure to manage the rapport can be a serious bar to medical treatment. Therefore, in this study, a rapport management model shown in the following figure between doctors and patients in outpatient clinics is built on the above research findings. The following part goes to the explanation of Fig. 1.

Doctors may encounter patient problematic behavior, such as resisting the therapeutic alliance, which consumers an inordinate amount of clinician time [72] and threatens the doctor-patient bond. Given the doctor-patient interaction is deeply influenced by the relationship that develops over the course of treatment [73], the doctors’ rapport strategies towards patients, especially when patients press physicians’ buttons and display rapport-threatening acts, become particularly important. In line with RMM, the strategies employed by the physician in the collected data are divided into four sections, namely, illocutionary domain, discourse domain, participation domain and stylistic domain. In the illocutionary domain, compliments serve as the guardian and proponent of patients’ face, especially respectability face. Spencer [73] suggests that respectability face refers to the prestige, honor or “good name” that a person holds within a community. With respectability face, Ho [74] mentions that the bases of respectability face could be very different in various nations and social groups. Therefore, the term face covers situation-specific values, and attributes in specific social interactions. However, there is little research on face in medical consultations even it is an interesting social setting in which to investigate patients’ face in non-compliant encounters [75]. In this study, respectability face is identified as a significant element of rapport in medical consultations in terms of patients’ expectations. Similarly, equity rights offer fresh insights on medical consultations. Typically, equity rights from the equity principle uphold people’s independent construals of self [76]. However, the patients’ equity rights in joking have the new manifestation that doctors should look out for patients’ behavioral expectations and provide equitable care. Thus, joking leads to situations in which patients in conversations follow the doctors’ plans, and thus relationships with patients continue in the way they prefer. In the discourse domain, storytelling is the major strategy to build rapport. Compared with other studies, storytelling demonstrates the importance of emotional involvement more than interactional involvement. When the patients resist treatment recommendations, a story is an effective way of making them laugh and breaking the ice without raising anger in the rapport building process, which is seldom discussed in previous studies. As for the participation domain, the inclusion of people present, in other words, interactional involvement, has a significant influence on the rapport-building outcomes. The interactional involvement, grounded in the early work of Erving Goffman, describes the extent to which an individual participates in a common environment. The slightest untoward act can potentially tear a delicately woven social fabric within doctor-patient consultations. But the interactional involvement of doctors lacks attention and analysis, and this study regulates the flow of rapport-threatening events with doctors’ involvement in real-world examples, which is one of the pragmatic highlights. In the stylistic domain, the productive consultation is a testament that the frequent use of appropriate honorifics or titles is a stepping stone to a good rapport. As for types, titles are categorized into three types [77], including generic titles, kinship titles and occupational titles. The function of titles is usually explored within business interactions [78] rather than medical settings. However, titles are rarely discussed in the Chinese out-patient clinic. In this study, the rapport function of titles is demonstrated as a result of Chinese physicians’ efforts to introduce politeness into interactions with patients by using appropriate titles. In this study, the rapport function of titles is demonstrated as a result of physicians’ efforts to introduce into interactions with patients by using appropriate titles. Efforts within these domains are taken to combat threats to the rapport since rapport-threatening acts chip away at the rapport doctors are attempting to maintain. In addition, it is noted in the preveding chaart that pertinent clinical strategies are presented with concurrence. In part due to the fact that doctors frequently integrate multiple strategies to form a therapeutic alliance in this dynamic and then contribute to a harmonious doctor-patient relation. These strategies help the physicians to feel confident about tackling rapport-challenging behavior and motivated to boost overall patient wellness. In the best case, their engagement in consultations creates a virtuous cycle of building a harmonious physician-patient relationship. It is the time that the expected medicine effect, lessened symptoms and improved health outcomes fell into place.

## Conclusion

The present study provides insights into physicians’ rapport-building strategies in the face of rapport-threatening behavior from patients. Specially, the study has shed light on the physician-patient relation based on RMM, which is a novel perspective that is very likely to see more in the future, considering that the physician-patient communication is important in the delivery of high-quality health care. The therapeutic effect of a physician’s treatment depends heavily on the trust and cooperation of their patients, making rapport-building a crucial skill. Recognizing the significance of a good rapport with patients during medical consultations has become commonplace among physicians. In this study, we offer broader insights into Rapport Management Model to explore physicians’ strategies in out-patient clinics. The study shows how physicians simultaneously build rapport and fulfill duties in the face of rapport-threatening behavior from patients. In particular, the inclusion of four domains, namely the illocutionary domain, the discourse domain, the participation domain and the stylistic domain, gives new insights into the delivery of high-quality health care. In the illocutionary domain, physicians can pay patients proper compliments to boost their confidence and make patients feel comfortable when patients decline the treatment recommendations. However, using compliments is not a surefire plan because some patients can be easily embarrassed by compliments [79]. Even if patients initially exhibit treatment resistance, joking can be the icebreaker to convey treatment plans and health education in a relaxing way. In the discourse domain, storytelling motivates patients to tell and retell to reestablish connections with doctors and empathy as a byproduct also strikes this chord. Furthermore, joint participation, especially the physicians’ participation also promotes patients to take the medicine. Additionaly, the genre-appropriate titles bring doctors and patients closer and cement their bonds. These findings demonstrate the practical implementation of rapport management strategies for doctors when they face patients’ rapport-threatening behavior in out-patient clinics. Four major pragmatic effects are achieved through doctors’ speech, including the face-work, especially the respectability face, the equity rights, the emotional involvement, the interactional involvement and the politeness consideration.

Considering that the physician-patient communication is important in the delivery of high-quality health care, the study takes a pragmatic approach to unpacking these rapport strategies and sheds light on the physician-patient relation, which is a new perspective that is very likely to see more in the future. There are many different directions for future work on rapport building to take. The limitation of this study is that the non-verbal strategies are not discussed because of the limited access to data. Thus, the combination of both audio and video data will provide a more diverse perspective to systematically study strategies for rapport building. Currently, physicians and patients are expected to engage in collaborative rapport management, achieving their agreed upon healthcare goals. The rapport management presented here represents merely a fraction of potential research waiting to be explored concerning rapport-building strategies in the medical setting. It is hoped that broad rapport-building strategies between physicians and patients can be extended or contested in future research.

## Data Availability

The datasets analyzed during the current study are not publicly available due to confidentiality having been guaranteed to the participants.. However, data are available from the authors upon reasonable request, conditioned by the permission of the hospital and participants. If someone wants to request the data from this study, please contact the corresponding author, SU Yonggang, syg@sdu.edu.cn.
